# Asymmetric Dimethylarginine, Endothelial Nitric Oxide Bioavailability and Mortality in Sepsis

**DOI:** 10.1371/journal.pone.0017260

**Published:** 2011-02-18

**Authors:** Joshua S. Davis, Christabelle J. Darcy, Tsin W. Yeo, Catherine Jones, Yvette R. McNeil, Dianne P. Stephens, David S. Celermajer, Nicholas M. Anstey

**Affiliations:** 1 International Health Division, Menzies School of Health Research and Charles Darwin University, Darwin, Northern Territory, Australia; 2 Division of Medicine, Royal Darwin Hospital, Darwin, Northern Territory, Australia; 3 Department of Medicine, University of Sydney and Department of Cardiology, Royal Prince Alfred Hospital, Sydney, New South Wales, Australia; 4 Intensive Care Unit, Royal Darwin Hospital, Darwin, Northern Territory, Australia; Leiden University Medical Center, Netherlands

## Abstract

**Background:**

Plasma concentrations of asymmetric dimethylarginine (ADMA), an endogenous inhibitor of nitric oxide synthase, are raised in patients with chronic vascular disease, causing increased cardiovascular risk and endothelial dysfunction, but the role of ADMA in acute inflammatory states is less well defined.

**Methods and Results:**

In a prospective longitudinal study in 67 patients with acute sepsis and 31 controls, digital microvascular reactivity was measured by peripheral arterial tonometry and blood was collected at baseline and 2–4 days later. Plasma ADMA and L-arginine concentrations were determined by high performance liquid chromatography. Baseline plasma L-arginine: ADMA ratio was significantly lower in sepsis patients (median [IQR] 63 [45–103]) than in hospital controls (143 [123–166], p<0.0001) and correlated with microvascular reactivity (r = 0.34, R^2^ = 0.12, p = 0.02). Baseline plasma ADMA was independently associated with 28-day mortality (Odds ratio [95% CI] for death in those in the highest quartile (≥0.66 µmol/L) = 20.8 [2.2–195.0], p = 0.008), and was independently correlated with severity of organ failure. Increase in ADMA over time correlated with increase in organ failure and decrease in microvascular reactivity.

**Conclusions:**

Impaired endothelial and microvascular function due to decreased endothelial NO bioavailability is a potential mechanism linking increased plasma ADMA with organ failure and death in sepsis.

## Introduction

Asymmetric dimethylarginine (ADMA), an endogenous non-specific nitric oxide synthase (NOS) inhibitor, is associated with chronic endothelial dysfunction [1] and increased cardiovascular risk [2], but its role in the setting of acute infections has been less well characterised.

Severe sepsis (acute infection resulting in organ dysfunction) is the leading cause of death in intensive care units in the USA [3], and is increasing in incidence globally [4]. Microvascular and endothelial dysfunction are key contributors to organ failure and death in sepsis but the mechanisms linking sepsis with vascular dysfunction remain incompletely understood [5]. A relative deficiency of constitutively expressed endothelial nitric oxide (NO), essential to maintain a quiescent and functional endothelium, may underlie sepsis-associated endothelial and microvascular dysfunction [6], [7]. NO is produced by NOS from its primary substrate, L-arginine. ADMA competitively inhibits the production of NO by NOS and additionally, along with symmetrical dimethylarginine (SDMA) and L-lysine, competes with L-arginine for transport across the cell membrane [8]. Hence the L-arginine: ADMA ratio is considered a better indicator of the availability of L-arginine to NOS than is plasma L-arginine concentration alone [9].

Infusion of ADMA in both rats [10] and humans [11] acutely decreases NO production, resulting in endothelial dysfunction. Plasma ADMA concentrations are increased in patients with chronic renal disease [12], hypertension [13], diabetes mellitus [14] and peripheral vascular disease [15]. Furthermore, ADMA has been shown to be an independent predictor of cardiovascular events in patients with existing coronary artery disease [16] and end-stage renal disease [17].

In contrast, few studies have examined the role of ADMA in humans with sepsis, and none have reported L-arginine: ADMA ratios or examined microvascular reactivity in this context. The few clinical studies that have reported plasma ADMA concentrations during acute infection have had conflicting results [18], [19], [20], [21]. Using peripheral arterial tonometry, we have previously shown that digital microvascular reactivity, a measure of endothelial NO bioavailability [22], is decreased in patients with sepsis [7]. However, we did not find a correlation between concentrations of plasma L-arginine and microvascular reactivity. We also found that despite an increase in plasma L-arginine concentrations over time, there was no corresponding improvement in microvascular reactivity. A potential explanation for these findings in sepsis is competitive inhibition of NOS by ADMA.

We hypothesised that plasma L-arginine: ADMA ratio would be decreased in sepsis, in proportion to disease severity, and would correlate with reactive hyperaemia peripheral arterial tonometry (RH-PAT) index, an *in vivo* measure of endothelial NO bioavailability. Furthermore, we hypothesised that increased plasma ADMA would be associated with mortality.

## Methods

### Study design and setting

We performed a prospective observational study at a 350-bed Australian teaching hospital, with an 18-bed mixed intensive care unit (ICU). Approval was obtained from the Human Research Ethics Committee of the Menzies School of Health Research and the Department of Health and Community Services. Written informed consent was obtained from all participants or next of kin where necessary.

### Participants

The study subjects were adults (≥18 years) hospitalised with sepsis, who were enrolled in a previously-reported study of microvascular reactivity; more detail of subject recruitment and study procedures are provided in this paper [7]. Sepsis was defined as a proven or suspected infection plus at least 2 criteria for the systemic inflammatory response syndrome (SIRS) present within the last 4 hours [23]; these include tachycardia (heart rate>90 beats per minute), tachypnoea (respiratory rate >20 breaths/minute), abnormal temperature (body temperature >38°C or <36°C), and abnormal white blood cell count (<4,000 cells/ml or >12,000 cells/ml or >10% band forms). Septic patients were eligible for enrolment within 24 hours of their admission to the ICU, or within 36 hours of admission to the ward. Control subjects were adults recruited from hospitalised patients with no clinical or laboratory evidence of inflammation or infection, and who had not met SIRS criteria within the last 30 days. Septic patients were classified as septic shock, or sepsis without shock. Septic shock was defined at the time of enrolment as systolic blood pressure <90 mmHg or a reduction of ≥40 mmHg from baseline despite adequate fluid resuscitation, or the need for vasopressors to maintain these targets [23]. Disease severity was assessed by the Acute Physiology and Chronic Health Evaluation (APACHE) II score and organ failure was determined using the Sequential Organ Failure Assessment (SOFA) score [24].

### Laboratory assays

Blood from arterial lines if present, or venepuncture if not, was collected in lithium heparin tubes at baseline and 2–4 days later, and plasma was separated and stored at −70°C within 2 hours of blood collection. Control patients had blood collected at baseline only.

ADMA and SDMA were measured by reverse phase HPLC with simultaneous fluorescence and UV-visible detection, as previously described [25]. The method precision, represented by percent relative standard deviation was 2.0% for ADMA and 2.3% for SDMA. Method accuracy measured by percent spike recovery was 98% for ADMA and 99% for SDMA. Arginine was measured using a method modified from van Wandelen and Cohen [26]. Angiopoietin-2 (Ang-2) and intracellular adhesion molecule-1 (ICAM-1) were measured by ELISA (R&D systems). IL-6 and TNFα were measured by flow cytometry using a cytokine bead array (BD Biosciences, CA, USA).

### Measurement of microvascular reactivity

Microvascular reactivity was measured at the bedside by RH-PAT (Itamar Medical, Caesarea, Israel), a non-invasive method of assessing endothelial function [27]–[28] which is at least 50% dependent on endothelial NO production [22]. Peripheral arterial tonometry (PAT) was measured in a fingertip before and after a 5-minute ischemic stress at the forearm, generating an RH-PAT index, normalized to the control arm, as previously reported [7].

### Statistical methods

Continuous variables were compared using Mann Whitney U test, and categorical variables using Fisher's exact test. Correlates with baseline ADMA and arginine:ADMA ratio were determined using Spearman's coefficient for univariate analysis. Day 2 values were compared with baseline values using paired Wilcoxon signed-rank test. In an *a priori* analytical plan, the relationship between baseline ADMA and mortality among sepsis patients was examined using logistic regression, with ADMA divided into quartiles as previously described [29]. To examine longitudinal correlations, linear mixed-effects models were used. A 2-sided p-value of <0.05 was considered significant. All analyses were performed using Intercooled Stata 10 (Statacorp, Texas).

## Results

There were 20 subjects with septic shock, 47 with sepsis without shock and 31 controls. The three groups were well-matched in terms of age, sex and known associations with chronically raised ADMA (**Table 1**).

**Table 1:** Baseline characteristics.

	*Septic Shock*	*Sepsis without shock*	*Controls*	*p value* a
**n**	20	47	31	
**Age** b	51.5(12.0)	52.5 (14.4)	45.4 (12.7)	NS
**Male** c	11 (55)	30 (63)	24 (75)	NS
**Diabetic** c	6 (30)	13 (27)	10 (31)	NS
**Smoker** c	8 (40)	22 (46)	14 (44)	NS
**IHD** c	4 (20)	8 (17)	4 (13)	NS
**Hypertension** c	5 (25)	17 (35)	9 (28)	NS
**Hyperlipidemia** c	4 (20)	11 (22)	11 (34)	NS
**Chronic renal disease** c	4 (20)	4 (8)	3 (10)	NS
**APACHE II score** d	20.0 (16–23)	10.0 (6–16)		<0.0001
**SOFA score** d	6 (3–9)	2.0 (0.5–4.0)		<0.0001

a – by Chi^2^ test for difference between all 3 groups.

b – Mean (sd).

c – n (%).

d – Median (Interquartile range).

### Arginine:ADMA ratio and disease severity

**Figure 1:**
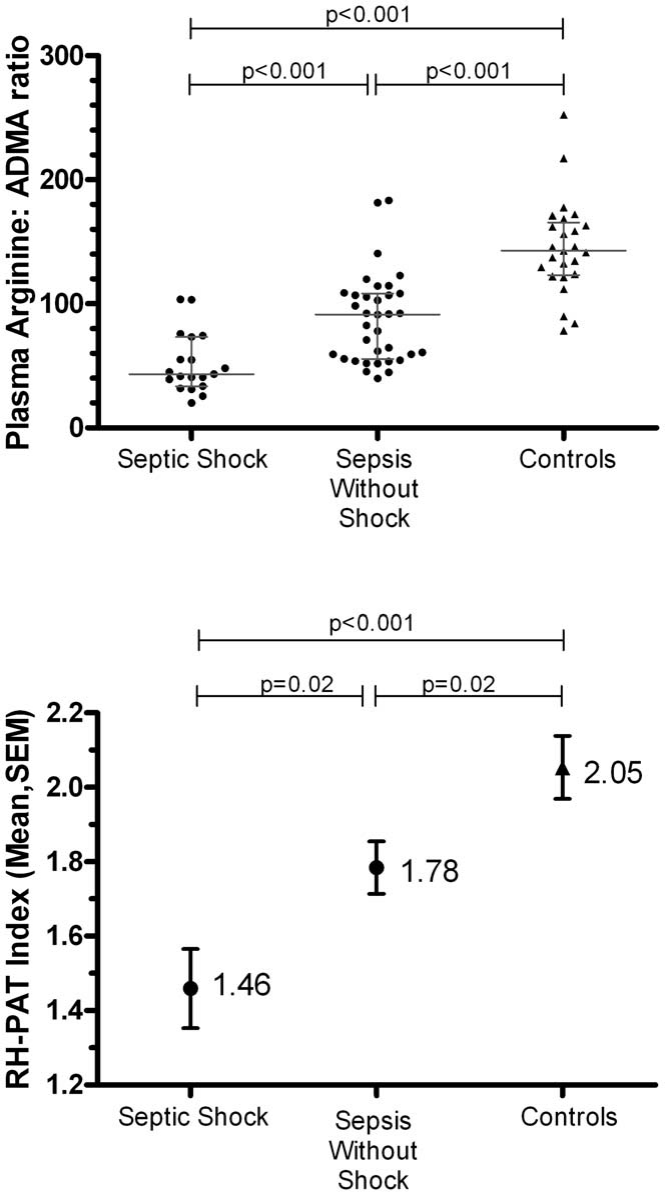
Ratio of L-arginine to asymmetric dimethylarginine in baseline plasma samples, according to disease category, compared with baseline microvascular reactivity according to disease category. Panel A shows plasma arginine: ADMA ratio and panel B shows reactive hyperaemia peripheral arterial tonometry index. P values represent comparisons between groups. Solid circles represent individual sepsis subjects and solid triangles represent individual control subjects. Horizontal lines represent median group values, and error bars represent interquartile range. In panel B, solid circles represent mean group values for sepsis subjects, and the solid triangle for control subjects. Error bars represent standard error of the mean.

Baseline plasma L-arginine: ADMA ratio was significantly lower in sepsis patients (median [IQR] 63 [45–103]) than in hospital controls (143 [123–166], p<0.0001) **(** **Table 2** **)**. Furthermore, septic shock patients had significantly lower L-arginine: ADMA ratio (median [IQR] 43 [34–73]) than sepsis patients without shock (91 [56–108], p<0.0001) (**Figure 1a**). The plasma L-arginine: ADMA ratio inversely correlated with severity of illness as measured by APACHE II score (r = −0.4, R^2^ = 0.16, p = 0.003) and organ failure as measured by SOFA score (r = −0.5, R^2^ = 0.25, p = 0.0001).

**Table 2:** Plasma asymmetric dimethylarginine and related variables at time of initial measurement.

	*All sepsis*	*Septic shock*	*Sepsis without shock*	*Control*	*p value pooled sepsis v control*	*p value septic shock vs control*
**n**	67	20	47	31		
**Plasma ADMA(µmol/L)** a	0.52 (0.39–0.65)	0.64 (0.54–0.85)	0.47 (0.38–0.57)	0.57 (0.50–0.62)	0.10	0.09
**Plasma L-arginine (µmol/L)** a **,** b	35.5 (27.3–51.2)	31.0 (23.7–40.4)	38.1 (29.4–51.7)	81.8 (68.9–91.3)	<0.001	<0.001
**Plasma L-arginine/ADMA ratio** a **,** b	63.2 (45.3–103.4)	43.4(33.6–73.3)	91.4 (55.5–108.3)	142.9 (123.0–165.7)	<0.001	<0.001
**Plasma SDMA(µmol/L)** a	0.66 (0.50–1.29)	1.05 (0.77–1.45)	0.56 (0.45–0.80)	0.47 (0.43–0.65)	0.002	<0.001
**Plasma lysine(µmol/L)** a	128 (100–171)	129 (90–190)	128 (104–162)	184 (157–216)	<0.001	0.006
**Receiving mechanical ventilation** c	14 (21)	9 (47)	5 (26)	-	-	-
**RH-PAT index** d	1.70 (0.47)	1.47 (0.40)	1.78 (0.47)	2.05 (0.46)	0.001	<0.001
**Plasma Interleukin 6 (pg/ml)** a	223 (76.6–563)	885 (298–2412)	148 (46.0–322)	4.7 (2.2–9.5)	<0.001	<0.001
**White blood cell count** a	15.2 (10.1–20.2)	17.5 (11.0–27.8)	15.2 (9.1–17.8)	7.7 (5.7–9.0)	<0.001	<0.001
**C-reactive protein** a	180 (87.3–259)	202 (126–297)	143(84–259)	7 (4–22)	<0.001	<0.001

a. median (Interquartile range);

b. n = septic shock 19, sepsis without shock 37, controls 27;

c. n (%).

d. mean (sd).

### ADMA, disease severity and mortality

The median [IQR] plasma concentration of ADMA was significantly higher in septic shock patients (0.64 [0.54–0.85] µM) than sepsis patients without shock (0.47 [0.38–0.57] µM) (p = 0.008) (**Table 2**) and correlated with SOFA score (r = 0.45, R^2^ = 0.20, p<0.001). Six of 67 sepsis patients (9%) had died by day 28 of follow-up, 5 of whom were in the septic shock subgroup. Median [IQR] baseline ADMA was approximately twice as high in those who died (1.07 [0.75–1.31]) as in survivors (0.51 [0.39–0.61]), p = 0.001. Sepsis patients with a baseline plasma ADMA concentration in the highest quartile (≥0.66 µmol/L) had an odds ratio for death of 20.8 (95% CI 2.2–195.0, p = 0.008). In a multivariate model incorporating SOFA score, age, gender, creatinine and IL-6 concentration, baseline ADMA was the only significant predictor of death (p = 0.04).

### SDMA, renal function and disease severity

SDMA was highest in septic shock, intermediate in sepsis without shock and lowest in controls (**Table 2**). Predominantly renally excreted [30], SDMA correlated strongly with serum creatinine (r = 0.70, R^2^ = 0.49, p<0.001), whereas ADMA did not (r = 0.16, R^2^ = 0.03, p = NS). On univariate analysis, sepsis patients with a plasma SDMA concentration in the highest quartile (≥1.30 µmol/L) had an odds ratio for death of 8.12 (95% CI 1.33–50.0), however this became insignificant on controlling for renal function.

### Arginine, ADMA and microvascular reactivity

There was a modest but significant correlation between baseline L-arginine: ADMA ratio and NO-dependent microvascular reactivity as measured by RH-PAT (**figures 1a and 1b**) both on univariate analysis (r = 0.34, R^2^ = 0.12, p = 0.02), and in a multivariate linear regression model adjusting for serum creatinine (Wald p-value for L-arginine: ADMA ratio = 0.03). The L-arginine: ADMA ratio was significantly lower in sepsis patients who required vasopressors (median [IQR]  = 42 [32–55]) compared to those who did not (74 [54–108], p = 0.002). Baseline plasma ADMA concentration correlated with markers of endothelial activation including Ang-2 (r = 0.45, R^2^ = 0.20, p = 0.0002) and ICAM-1 (r = 0.47, R^2^ = 0.22, p = 0.0001). This relationship persisted after controlling for disease severity (using APACHE II score) in a multivariate analysis.

Over the first 2–4 days of follow up, plasma ADMA increased in the sepsis patients (0.53 to 0.64, p = 0.002) (**Table 3**), and also in the septic shock subgroup (0.64 to 0.85, p = 0.03). Plasma L-arginine concentrations also increased, but due to the increase in ADMA, there was no significant change in the L-arginine: ADMA ratio. In a mixed effects linear regression model examining change from baseline to day 2–4, increase in ADMA over time significantly correlated with increase in SOFA score (p<0.001) and decrease in RH-PAT index (p = 0.03), but not with change in IL-6 or CRP. It also correlated with increase in the liver (p<0.001) but not the renal (p = 0.09) components of the SOFA score.

**Table 3:** Longitudinal results in subjects with sepsis.

	Day 0	Day 2	P Day 0 to 2
**n**	67	47	
**ADMA**	0.53 (0.39–0.66)	0.64 (0.51–0.78)	0.002
**L-arginine**	35.5 (27.3–51.2)	47.2 (30.8–58.1)	0.03
**L-arginine: ADMA ratio**	63.2 (45.3–103.4)	63.0 (41.7–108.0)	NS
**RH-PAT index**	1.70 (1.57–1.82)	1.81 (1.65–1.96)	NS
**SDMA**	0.66 (0.50–1.30)	0.71 (0.47–1.36)	NS
**IL-6**	223 (78.2–530)	54.5 (16.1–201)	<0.001
**SOFA score**	3 (1–7)	2 (1–7)	0.04

Note: ADMA = Asymmetric dimethylarginine. RH-PAT index = Reactive hyperaemia peripheral arterial tonometry index. SDMA = Symmetric dimethylarginine. IL-6 = Interleukin 6. SOFA score = Sequential Organ Failure Assessment Score.

## Discussion

The plasma L-arginine: ADMA ratio is significantly reduced in sepsis, in proportion to disease severity. Plasma ADMA concentration correlates with the degree of organ failure and predicts mortality in patients with sepsis. Increase in ADMA over time is associated with worsening microvascular reactivity and organ dysfunction. Our results suggest a possible mechanism underlying these associations: impairment of microvascular function due to inhibition of endothelial NO production by ADMA.

Decreased L-arginine: ADMA ratio may contribute to organ failure in sepsis by reducing microvascular reactivity. Impaired microvascular and endothelial function have been shown to be important contributors to organ dysfunction and death in animals and humans with sepsis [31]. ADMA causes both acute [11] and chronic [2] endothelial dysfunction by inhibiting NOS and decreasing endothelial NO bioavailability. The L-arginine: ADMA ratio is a marker of the availability of L-arginine to NOS [9]. In severe malaria, plasma ADMA is increased and is associated with endothelial dysfunction and reduced exhaled nitric oxide [32]. In this study we found that baseline L-arginine: ADMA ratio, but not arginine or ADMA alone, correlated with endothelial nitric oxide dependent microvascular reactivity. Furthermore, plasma ADMA concentrations correlated with increased plasma concentrations of Ang-2 and ICAM-1, both of which are associated with reduced endothelial nitric oxide bioavailablity [7], [33], [34]. Together, these findings suggest that a decreased L-arginine: ADMA ratio reduces endothelial nitric oxide bioavailability and thus impairs microvascular reactivity in sepsis. This may provide a mechanistic explanation for the observed association of plasma ADMA concentrations with organ failure and death in this and other studies [20], [29]. However, although significant, this association was not strong and further work is needed to confirm this preliminary observation.

A recent small study in human volunteers injected with lipopolysaccharide also found an acute increase in plasma ADMA and decrease in NO-dependant vasodilatation, but did not find a correlation between NO-dependant vasodilatation and L-arginine:ADMA ratio [35]. These differing findings may be due to the fact that in Engelberger's study, the sample size (n = 7) was too small to detect such a correlation. In addition, sepsis is a highly complex pathophysiological state, and the findings from sepsis models may be difficult to apply to clinical sepsis.

The increase in plasma ADMA concentrations over time observed in this study agrees with the findings of another recent observational study in septic humans [21]. This increase may in part explain the lack of significant improvement in microvascular reactivity as patients recover [7], despite an increase in plasma L-arginine. This may be because the L-arginine: ADMA ratio (and thus the availability of L-arginine to NOS within endothelial cells) does not change over time. The mechanism behind the change in ADMA over time cannot be determined from these data, however there are several possibilities. Protein catabolism in patients with sepsis could lead to progressive release of methylated L-arginine residues into the plasma. However, this is unlikely to be the case because endogenous leucine flux (a measure of protein catabolism) does not correlate with plasma ADMA concentrations in septic humans [36]. NO causes direct inhibition of dimethylarginine dimethylaminohydrolase (DDAH) activity by S-nitrosylation of an active cysteine residue [37]. Thus it is possible that as patients recover from sepsis and endothelial NO bioavailability increases, DDAH activity is inhibited, resulting in an increase in plasma ADMA concentrations. Finally, the longitudinal inverse association between liver function and plasma ADMA suggests that worsening liver function due to sepsis progression, and thus decreased metabolism of ADMA, may also explain these findings.

The disparity between ADMA concentrations in shock and without shock may be due to different mechanisms within these two states. Early sepsis is a hyperdynamic state, with increased cardiac output and liver and kidney blood flow [38], [39]. This may lead to increased degradation of ADMA in the liver by DDAH and, to a lesser extent, increased renal excretion. This hypothesis is supported by a study which found that the liver fractional extraction rate for ADMA is significantly higher and circulating ADMA is significantly lower in endotoxemic rats compared to controls [40]. Patients with septic shock have generally developed multiple organ failure and down-regulation of cellular functions [41] and thus hepatic metabolism and renal excretion of ADMA may drop back to baseline concentrations. This hypothesis is supported by our finding that ADMA concentrations inversely correlate with liver function, both at baseline and longitudinally. Similar findings have recently been reported in patients with malaria, in whom plasma ADMA concentrations were raised in those with severe malaria but low normal in those with moderately severe malaria [32].

Our study helps to clarify the inconsistencies reported in previous clinical studies measuring ADMA in acute infections. It demonstrates that while patients with septic shock have increased ADMA, patients without shock have decreased ADMA resulting in no significant difference between the ADMA concentrations in pooled sepsis and hospital controls – a potentially misleading finding unless patients are stratified by sepsis severity. The previous studies that found that ADMA was increased in sepsis [18], [20], [21] primarily enrolled patients with septic shock. The only other published study to enrol sepsis patients without shock also found no overall difference in plasma ADMA concentrations between sepsis and control patients [19]; however, they did not consider patients with and without shock separately.

This study has several limitations. Although it is at least 50% dependant on endothelial NO [22], peripheral arterial tonometry is not a direct measure of NO activity. Other factors are likely to contribute to endothelial NO bioavailability in addition to the L-arginine:ADMA ratio, including CAT transport inhibitors (such as SDMA) and oxidative stress resulting in NO-quenching. The 67 sepsis patients were not all followed up on day 2-4, largely because of hospital discharge; thus the longitudinal results may underestimate the degree of improvement in microvascular and organ function.

Raised plasma ADMA concentrations are a strong predictor of death in patients with sepsis and thus may be useful as a prognostic marker. Impaired endothelial and microvascular function due to decreased endothelial NO production may be a mechanism linking ADMA with organ dysfunction and mortality. The DDAH-ADMA axis is a potential therapeutic target and may be important in individual tailoring of therapy. Agents which compete with ADMA for NOS (such as L-arginine) or which potentiate DDAH activity should be further investigated in sepsis.
